# Surgical Management of Mediastinal Ectopic Parathyroids

**DOI:** 10.3390/jpm15070276

**Published:** 2025-06-30

**Authors:** Giacomo Rabazzi, Gianmarco Elia, Vittorio Aprile, Stylianos Korasidis, Maria Giovanna Mastromarino, Diana Bacchin, Alessandra Lenzini, Marcello Carlo Ambrogi, Greta Alì, Filomena Cetani, Gabriele Materazzi, Marco Lucchi

**Affiliations:** 1 Department of Surgical, Medical and Molecular Pathology and Critical Care Medicine, University of Pisa, 56124 Pisa, Italy; giacomo.rabazzi@gmail.com (G.R.); gabriele.materazzi@unipi.it (G.M.); marco.lucchi@unipi.it (M.L.); 2 Division of Thoracic Surgery, Cardiac, Thoracic and Vascular Department, University Hospital of Pisa, Via Paradisa 2, 56100 Pisa, Italymgmastromarino@gmail.com (M.G.M.);; 3 Unit of Endocrinology, Department of Clinical and Experimental Medicine, University of Pisa, 56124 Pisa, Italy

**Keywords:** ectopic parathyroid glands, hyperparathyroidism, thoracic surgery, minimally invasive surgery

## Abstract

Primary hyperparathyroidism is commonly caused by parathyroid adenomas, hyperplasia, or, rarely, carcinoma. In up to 20% of cases, parathyroid tissue may be ectopic, often located in the mediastinum due to aberrant embryologic migration. Ectopic parathyroid glands pose a diagnostic and therapeutic challenge, and an accurate preoperative localization is essential for an effective and safe resection. Imaging modalities such as CT scan, TC-sestamibi scintigraphy, PET/CT, ultrasonography and MRI are routinely employed, whereas combined techniques offer improved diagnostic accuracy. Emerging approaches, however, including PET/CT with choline tracers, have shown promise in enhancing sensitivity in complex or recurrent cases. When ectopic glands are in the mediastinum, thoracic surgical intervention is required. Traditional open approaches, such as sternotomy or thoracotomy, are associated with significant morbidity. The development and evolution of minimally invasive surgery (MIS) has become the preferred approach in selected cases. When MIS is performed, intraoperative assessment and parathyroid identification are crucial to ensure complete gland removal. Intraoperative parathyroid hormone (ioPTH) monitoring provides real-time confirmation of surgical success. The integration of advanced imaging, intraoperative monitoring, and minimally invasive techniques significantly improves surgical outcomes while minimizing complications and accelerating patient recovery. Ultimately, the effective treatment of ectopic parathyroid glands relies on a personalized approach, adapting both diagnostic and surgical strategies to the unique anatomical and clinical context of each patient. Integration of advanced imaging, intraoperative monitoring, and minimally invasive techniques, combined with a multidisciplinary team involving endocrinologists, radiologists, and thoracic surgeons, is key to optimizing outcomes and reducing patient morbidity.

## 1. Introduction

Hyperparathyroidism is a medical condition characterized by the excessive secretion of parathyroid hormone (PTH), which leads to a wide range of systemic effects [1]. PTH has a regulatory role in calcium and phosphate metabolism; therefore, its imbalance can have a multi-organ impact, affecting the bones, kidneys, gastrointestinal system, and neuromuscular function. There are two common forms of this condition, primary hyperparathyroidism (PHPT) and secondary hyperparathyroidism (SHPT), while tertiary hyperparathyroidism (THPT) is relatively rare.

In PHPT, PTH is overproduced, leading to elevated serum calcium (sCa) levels—a condition known as hypercalcemia. The most frequent underlying cause is solitary parathyroid adenoma, which is responsible for approximately 85% of cases. Less commonly, the condition may be caused by parathyroid hyperplasia involving multiple glands or, rarely, parathyroid carcinoma [2].

Prolonged elevations in serum calcium can lead to progressively worsening bone resorption, vascular calcification, nephrolithiasis, and other complications, highlighting the importance of timely diagnosis and management [3].

Secondary hyperparathyroidism, on the other hand, is usually a consequence of chronic kidney disease or after kidney transplantation, particularly in individuals with end-stage renal disease (ESRD). In this setting, impaired phosphate excretion, reduced vitamin D activation, and hypocalcemia stimulate a compensatory overproduction of PTH. Unlike PHPT, SHPT is characterized by diffuse hyperplasia of all four parathyroid glands rather than a single adenomatous lesion.

Tertiary hyperparathyroidism, finally, is characterized by autonomous and excessive secretion of parathyroid hormone (PTH) following prolonged secondary hyperparathyroidism, commonly observed in patients with chronic kidney disease. This condition leads to persistent hypercalcemia despite correction of the initial stimulus.

The majority of hyperfunctioning parathyroid glands are in the cervical region, typically close to the thyroid gland, reflecting their normal embryologic development. However, in approximately 15–20% of cases, these glands are found in ectopic locations due to aberrations in embryological migration. Ectopic parathyroid tissue can be located anywhere along the path of descent from the third and fourth pharyngeal pouches, including sites such as the thymus, carotid sheath, retroesophageal space, and mediastinum. Among these ectopic cases, approximately 1–3% are situated deep within the thoracic cavity, particularly the anterior or posterior mediastinum, necessitating a thoracic surgical approach for successful resection [4,5].

Parathyroidectomy remains the only curative treatment for primary hyperparathyroidism. Surgical intervention is indicated in patients with symptomatic disease, such as those presenting with hypercalcemia-related symptoms (e.g., fatigue, neurocognitive dysfunction), nephrolithiasis, or skeletal involvement like osteoporosis or fragility fractures. It is also recommended in asymptomatic individuals who meet specific biochemical thresholds, such as a serum calcium concentration persistently greater than 1 mg/dL above the upper limit of normal, or markedly elevated parathyroid hormone (PTH) levels in the presence of end-organ effects, including reduced bone mineral density or impaired renal function [6].

The management of mediastinal ectopic parathyroid glands presents significant clinical challenges due to their complex anatomical relationships with vital thoracic structures, including the great vessels (such as the aorta and pulmonary artery), the trachea, esophagus, and pericardium. These anatomical intricacies necessitate individualized preoperative planning and a flexible diagnostic strategy. Precise imaging localization, using a combination of traditional, hybrid, and emerging modalities, must be tailored case by case to define the safest and most effective surgical approach, while minimizing the risk of complications. This review aims to provide a comprehensive overview of the current state of knowledge on mediastinal ectopic parathyroids, emphasizing the importance of personalized diagnostic and surgical strategies. It discusses diagnostic pathways, operative techniques, and postoperative considerations, integrating insights from recent literature to highlight the need for a patient-specific management paradigm.

## 2. Preoperative Identification Strategies

**Figure 1:**
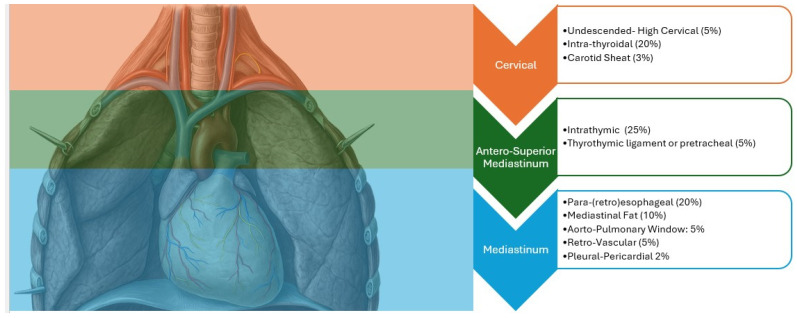
This image illustrates the ectopic locations of parathyroid glands, categorized anatomically into three main regions: cervical, antero-superior mediastinum, and mediastinum.

Due to the embryological origin of the parathyroid glands and the laxity of mediastinal tissues, which do not impose anatomical constraints on their migratory potential, a parathyroid gland can be located virtually anywhere within the mediastinum. Nevertheless, certain mediastinal sites exhibit a higher predilection for harboring hyperfunctioning parathyroid glands. These include locations within the thymus, adjacent to the thoracic esophagus, near major vessels (such as the ascending aorta, aortic arch, aortopulmonary window, and superior vena cava), and overlying the pericardium [7] as reported in Figure 1. Accurate preoperative localization is therefore critical for minimizing surgical morbidity and avoiding unnecessary thoracic exploration [8].

Currently, preoperative localization encompasses a variety of techniques, ranging from advanced imaging modalities to selective venous sampling for elevated parathyroid hormone (PTH) levels. The choice of localization strategy depends on institutional expertise and resource availability, and as such, existing guidelines are intentionally flexible. Nevertheless, the use of nuclear imaging techniques ubiquitously constitutes a cornerstone in diagnostic standards, regardless of the local context. Imaging techniques have evolved substantially, offering high sensitivity and specificity in detecting ectopic parathyroid tissue. Commonly used modalities include ultrasonography, computed tomography (CT), magnetic resonance imaging (MRI), positron emission tomography (PET), and Technetium-99m sestamibi (99mTc-sestamibi) scanning [7]. However, the diagnostic performance of each modality varies depending on the clinical context [9], making it essential for thoracic surgeons to understand the strengths and limitations of each in order to tailor the diagnostic work-up to the individual patient and avoid redundant investigations.

**Figure 2:**
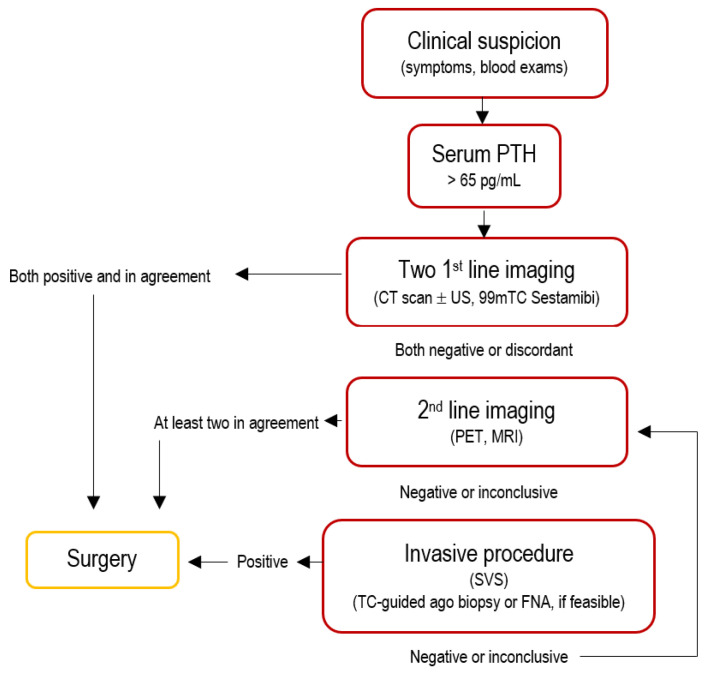
This flowchart outlines a diagnostic algorithm for primary hyperparathyroidism based on imaging and serum parathyroid hormone (PTH).

A combination of neck ultrasonography and 99mTc-sestamibi scintigraphy or four-dimensional computed tomography (4D-CT) is recommended as the most cost-effective first-line approach for localizing parathyroid adenomas, with each modality serving a complementary rather than competitive role. Second- and third-line imaging modalities—including MRI and PET/CT—are typically reserved for challenging cases, redo surgery, or when first-line methods are contraindicated, as reported in Figure 2.

### 2.1. Computed Tomography

Computed tomography (CT), particularly four-dimensional CT (4D-CT), is typically performed from the mandible to the mediastinum—especially when a neck ultrasound (US) has not been conducted or has yielded suspicious or negative findings—in order to identify hyperfunctioning parathyroid glands. Four-dimensional CT employs multiphase imaging (non-contrast, early post-contrast, and delayed post-contrast phases), where the “fourth dimension” refers to time, to differentiate abnormal parathyroid tissue based on its characteristic enhancement pattern: a brisk contrast uptake (blush) followed by early washout [10]. This modality has shown high accuracy, exceeding 85%, in localizing ectopic parathyroid glands [6,11,12].

Larger parathyroid adenomas or hyperplastic glands are generally well visualized with CT; however, smaller lesions can be more difficult to detect. Additionally, reactive lymph nodes can mimic hyperfunctioning parathyroid tissue, as their enhancement and washout patterns may be similar, leading to potential false positives. One notable limitation of 4D-CT is the relatively high radiation dose—up to four times that of standard CT scans. Furthermore, the requirement for iodinated contrast may pose a risk to patients with contrast allergies or renal insufficiency. This is particularly relevant in individuals with hyperparathyroidism, as disturbances in calcium metabolism can contribute to compromised renal function.

### 2.2. Tc-99m Sestamibi Scintigraphy

Technetium-99m sestamibi scintigraphy remains, to date, the most widely used imaging modality for localizing hyperfunctioning parathyroid tissue [13]. All patients should undergo a sestamibi nuclear medicine scan, which includes at least one of the following: planar scintigraphy, single-photon emission computed tomography (SPECT), or SPECT combined with computed tomography (SPECT/CT). This technique is based on the principle that sestamibi accumulates within the mitochondria of hypermetabolic cells, including those of ectopic parathyroid glands.

The imaging is typically performed in two phases—early (5–15 min) and delayed (2–4 h) after intravenous administration of the radiotracer—targeting both the anterior neck and chest regions using a pinhole collimator. Parathyroid adenomas generally exhibit focal radiotracer uptake during the early phase, which persists during delayed imaging. The intensity of the signal correlates with both the size and metabolic activity of the abnormal parathyroid tissue. As a result, this technique effectively identifies only hyperfunctioning glands, thereby helping to differentiate them from normal parathyroid tissue. However, the sensitivity of 99mTc-sestamibi is reduced in the presence of small (<600 mg) or moderately to poorly functioning adenomas, particularly those with a low percentage of oxyphilic cells (<20%) [1]. The sensitivity of 99mTc-sestamibi scintigraphy is reported to range between 80% and 90% for single adenomas, but its accuracy declines in cases of multiglandular disease or parathyroid hyperplasia [14]. False positives can occur; for instance, thymomas often show increased sestamibi uptake due to their high mitochondrial content—a confounding factor, given that the thymus is a frequent site of ectopic parathyroid glands [15,16]. Additionally, the thyroid gland can also take up 99mTc-sestamibi, underscoring the need to exclude the presence of ectopic thyroid tissue in the mediastinum prior to surgery [14,17].

Although nuclear medicine techniques are burdened with a lower level of administered radiation compared to 4D-CT, 99mTc-sestamibi scanning is limited by its relatively poor spatial resolution, particularly in the mediastinum. This can make precise localization difficult, especially when ectopic parathyroid tissue is adjacent to other structures such as lymph nodes, which may not capture the tracer but can obscure the signal.

Although the combination of cervical ultrasonography and [99mTc] Tc-MIBI scintigraphy is widely used, 4D-CT may be useful in select cases, such as inconclusive or negative prior imaging, distorted neck anatomy, or after unsuccessful surgery, as stated in the EANM guidelines. Evidence suggests that concordant findings from two imaging modalities significantly reduce the likelihood of missing ectopic parathyroid tissue during surgery compared to relying on a single imaging technique [18]. This combined approach is especially valuable in cases where clinical signs of hyperparathyroidism are subtle and the ectopic gland is sub-centimetric in size, making it challenging to distinguish from nearby anatomical structures such as lymph nodes [11].

### 2.3. Single Photon Emission Computed Tomography

Single-photon emission computed tomography (SPECT) has been incorporated into the preoperative imaging workflow specifically to address the limited spatial resolution of planar 99mTc-sestamibi scintigraphy. When used in combination, 99mTc-sestamibi SPECT significantly enhances diagnostic accuracy by providing three-dimensional localization of hyperfunctioning parathyroid tissue. Sensitivity can reach up to 96%, and specificity is also markedly improved compared to planar imaging alone [19,20,21]. However, studies suggest that SPECT is less effective at detecting hyperplastic glands compared to solitary adenomas, likely due to the lower metabolic activity and smaller size of hyperplastic tissue [14]. In the absence of SPECT, a useful alternative is the use of three-dimensional multiplanar reconstructions from standard CT scans, which may help compensate for the lack of functional imaging data.

Finally, 99mTc-sestamibi SPECT can be integrated with CT to form a hybrid SPECT/CT modality. This combined approach merges functional (sestamibi uptake) and anatomical (CT morphology) information and is often sufficient as a standalone diagnostic tool. However, if SPECT/CT results are inconclusive or negative, the value of additional localization studies—such as high-resolution 4D-CT or MRI—increases significantly to ensure accurate surgical planning.

### 2.4. Neck Ultrasound

Cervical ultrasonography has limited utility for detecting mediastinal lesions due to acoustic shadowing and interference from overlying bony and aerated structures. Nonetheless, recent evidence has highlighted a renewed role for ultrasound in the preoperative setting. A large study involving over 700 patients demonstrated that combining neck ultrasound with 99mTc-sestamibi SPECT/CT resulted in greater sensitivity than SPECT/CT alone [22].

Once mediastinal parathyroid tissue is suspected or confirmed, a thorough ultrasound evaluation of the neck should always be performed—if not already completed—to rule out the presence of additional ectopic or hyperfunctioning parathyroid tissue in the cervical region. Ultrasonography remains a non-invasive, cost-effective, and radiation-free modality, with reported sensitivities ranging from 72% to 89% in the detection of single-gland disease [23].

### 2.5. Magnetic Resonance Imaging (MRI)

Also, magnetic resonance imaging (MRI) could have a role, as parathyroid adenomas have intermediate to low signal intensity on T1-weighted images and high signal intensity on T2-weighted images. However, signal intensity characteristics are not unique to parathyroid adenomas, so their use is still limited in patients with persistent cervical disease, and as a confirmatory technique to be used in the second instance or where more traditional techniques would not prove decisive results. If necessary, its sensitivity can be improved with contrast-enhanced multiparametric dynamic MRI, exploiting the hypervascular nature of adenomas. Lastly, MRI may assist the clinician in assessing the relationship of parathyroid adenomas with adjacent structures, particularly in cases of adherence to or infiltration of major blood vessels, neural structures, or the airway and digestive tract.

### 2.6. Positron Emission Tomography (PET/CT)

More recently, positron emission tomography (PET/CT) has emerged as a promising modality for cases where traditional imaging fails, demonstrating superior sensitivity in detecting hyperfunctional parathyroid tissue, as long as PET offers greater spatial resolution than SPECT [24,25]. Both [¹⁸F]fluorocholine and [¹¹C]choline can be used as tracers with high sensitivity (95%) results [26]. Later studies have been so promising that investigators concluded that choline PET/CT should be the modality of choice for initial localization [27,28]. An alternative radiotracer is [¹¹C]C-methionine, which has demonstrated a sensitivity of 80% in the detection of solitary parathyroid adenomas [29]. Durma et al. recently demonstrated a role for [¹¹C]C-MET PET/CT in patients with tertiary hyperparathyroidism who were resistant or intolerant to non-invasive treatments and had negative scintigraphy and neck ultrasonography results, reporting a sensitivity of 100% in patients subsequently undergoing surgery [30]. Further innovative methods include fusion of 4D CT with 18F-fluorocholine PET/CT and hybrid choline PET/MRI. However, the occurrence of two or more imaging tests that are inconsistent with each other is occasionally observed.

### 2.7. Pre-Operative Diagnosis

**Figure 3:**
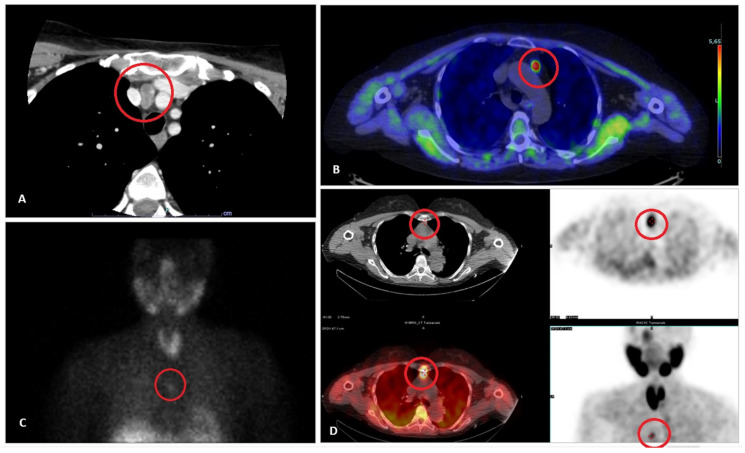
Representative radiological images of four different patients with ectopic parathyroid glands or adenoma trough different modalities: (**A**) Contrast-enhanced CT scan showing a lesion in the anterior mediastinum scan (circled); (**B**) ¹⁸F-fluorocholine PET/CT demonstrating focal radiotracer uptake consistent with hyperfunction-ing parathyroid tissue (circled); (**C**) 99mTc-sestamibi scintigraphy revealing increased uptake in the mediastinum (circled); (**D**) Multimodal imaging correlation (PET, CT, fused PET/CT, and maximum inten-sity projection) from a single patient confirming ectopic localization (circled).

The management of patients with ambiguous or discordant localization studies remains challenging [3], and most unsuccessful surgical explorations are characterized by this very issue [31]. When imaging results are inconclusive or conflicting, additional diagnostic approaches may be warranted, including invasive procedures such as selective venous sampling (SVS) or tissue biopsy, as reported in Figure 2 and Figure 3.

CT-guided needle biopsy is not routinely performed and is typically reserved for highly selected cases, particularly when the suspected lesion is located in the anterior mediastinum in close proximity to the chest wall and not obscured by bony structures, thus facilitating radiologic access. Alternatively, ultrasound-guided fine-needle aspiration (FNA) with parathyroid hormone (PTH) assay from the aspirate can be diagnostic in accessible lesions [23]. However, if malignancy is suspected, biopsy should be avoided due to the risk of tumor seeding.

Selective venous sampling, which measures PTH gradients across various venous sites, may be used in clinical practice to localize hypersecreting tissue, particularly in patients with persistent or recurrent hyperparathyroidism after prior surgery [32,33]. This procedure involves catheterization, usually via a transfemoral approach, to access the neck and mediastinum, during which up to 30 venous samples may be collected from various sites [19]. PTH concentrations from these samples are then compared with peripheral venous levels, and a twofold or greater increase is considered indicative of a positive localization.

Nonetheless, it is important to recognize that false positives can occur with SVS. Surgeons should exercise caution, particularly if SVS localizes a suspected gland to an area that appears radiologically normal on other imaging modalities.

## 3. Surgical Treatment

**Figure 4:**
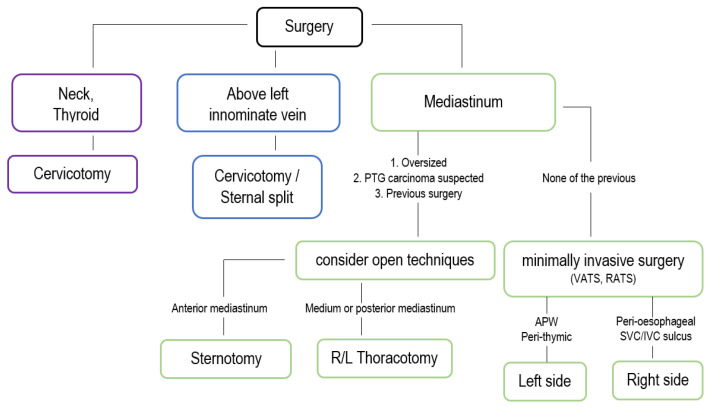
This flowchart delineates the selection process for the surgical approach to parathyroidectomy, guided primarily by the anatomical location of the parathyroid lesion and relevant clinical factors. It encompasses various thoracic surgical techniques, including transcervical approaches for lesions accessible via the neck, minimally invasive thoracoscopic surgery for lesions located within the upper mediastinum, and open thoracotomy reserved for deeply situated or complex mediastinal adenomas.

**Figure 5:**
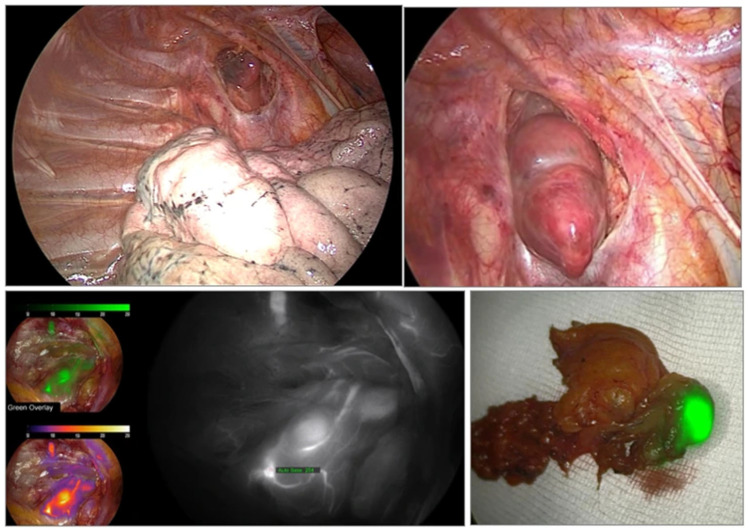
Intraoperative images of ectopic parathyroid glands located in the thorax, with demonstration of indocyanine green (ICG) fluorescence visualization both intraoperatively and after gland excision.

The gold standard treatment for hyperparathyroidism is surgical excision of the pathological tissue. Current guidelines recommend parathyroidectomy for asymptomatic patients based on age, serum calcium levels, bone density, and kidney function [34]. However, it is important to note that surgery should be considered for all PHPT patients, as it is the only curative option and is more cost-effective than pharmacological treatment or observation [35,36], as reported in Figure 4 and Figure 5.

Non-surgical options were investigated between the 1990s and 2000s, including angiographic ablation. In the literature, the use of various materials was reported, such as ionic contrast agents, alcohol, autologous clot, and silicone rubber, in order to obtain the occlusion of feeding vessels, typically a bronchial artery [37,38]. However, another study found that angioablation failed to control hyperparathyroidism in 40% of cases [39]; consequently, this technique has progressively become obsolete.

Currently, the surgical approach varies depending on the pathology to be treated. In cases of hereditary syndromes (e.g., MEN-1), two surgical approaches are recommended: subtotal parathyroidectomy, which involves the removal of most glands with partial in situ resection of the remaining one, and total parathyroidectomy, with or without heterotopic autotransplantation of a small portion of a gland into the brachialis muscle. Anderson et al. [40] reported no significant difference between these methods in terms of complication rates, readmission, or 30-day mortality. When patients are affected by parathyroid adenoma alone—cervical or intrathoracic—the excision of the affected gland, preserving the eutopic normally functioning glands in situ, may be sufficient.

### 3.1. Open Approaches

Traditional surgical techniques, such as median sternotomy and thoracotomy, were historically the standard approaches for deep-seated mediastinal parathyroid lesions [41,42,43]. However, advances in minimally invasive surgery have led to safer, more efficient, and less morbid procedures [44,45]. Median sternotomy offers excellent exposure to the central mediastinum and remains a valid option when complete excision is required or when multiple ectopic glands are suspected. Nevertheless, it is associated with significant morbidity, including longer recovery times and increased postoperative pain [3,46].

Thoracotomy, another traditional method, is selectively used for posterior mediastinal parathyroid adenomas but has fallen out of favor due to its invasiveness. The cervical approach is preferred when the ectopic gland is located in the upper mediastinum and can be accessed through a transcervical thymectomy. This approach is often effective for parathyroid glands within or adjacent to the thymus and minimizes the morbidity associated with thoracic access [47]. However, when parathyroid tissue is deeply situated below the aortic arch or located in the posterior mediastinum, a transthoracic approach becomes necessary.

Mediastinoscopy, though technically a transcervical approach, refers specifically to the anatomical plane anterior to the trachea and posterior to the great vessels. It is not typically performed by endocrine surgeons and plays a limited role in ectopic mediastinal PTGs, as fewer than 5% are found in this area [31]. Anterior mediastinotomy (parasternal or the Chamberlain approach) has been described [5], and upper partial sternotomy has been used to access challenging anatomical regions like the aortopulmonary window [48].

### 3.2. Minimally Invasive Approaches

Before 2010, sternotomy was the most frequently used approach. Even today, some authors [2,49] argue that it provides the best exposure and facilitates palpation of any additional mediastinal masses. However, despite the limited tactile feedback associated with minimally invasive techniques, video-assisted thoracoscopic surgery (VATS) and robot-assisted thoracoscopic surgery (RATS) have revolutionized the management of mediastinal parathyroid disease [50,51,52].

Introduced in the 1990s, VATS has become the preferred technique for mediastinal parathyroidectomy in appropriate patients. It offers several advantages over traditional open approaches, including reduced postoperative pain, shorter hospital stays, and lower complication rates [53,54]. The procedure involves the placement of small thoracic ports, allowing precise dissection and excision of the hyperfunctioning gland. Most mediastinal parathyroid lesions are located in the anterior mediastinum and are accessible via a left thoracoscopic approach, similar to thymectomy [15]. However, there is no clearly defined anatomical cutoff between VATS and cervical approaches. Iihara et al. [15] recommend a transcervical approach for lesions located at or above the aortic arch and a transthoracic approach for those located below.

In addition to lesion location, the choice of approach depends on patient preference and the surgeon’s experience with transcervical versus transthoracic thymectomy. RATS offers enhanced dexterity and visualization, making it a valuable alternative in complex cases. Studies have shown it to be feasible for mediastinal parathyroidectomy, particularly for lesions in difficult-to-access areas such as the aortopulmonary window or posterior mediastinum [55]. In line with other authors [44,56,57,58], Makey et al. advocate for a robotic transthoracic approach for lesions located within the thymus gland [31]. Nonetheless, despite these benefits, robot-assisted approaches are associated with longer operative times and higher costs, limiting their broader adoption.

### 3.3. Intraoperative Evaluation

To optimize surgical outcomes, intraoperative adjuncts such as rapid intraoperative parathyroid hormone (ioPTH) monitoring, meticulous tissue handling, and the use of adjunct imaging techniques are essential.

Nussbaum et al. [59] first described the use of intraoperative PTH measurements to confirm the adequacy of resection following the removal of a hyperfunctioning parathyroid gland. Intraoperative PTH monitoring is based on the short half-life of PTH (approximately 3–5 min), allowing for near-immediate assessment of surgical effectiveness within the anesthetic window. More than 90% of high-volume parathyroid surgeons in the United States utilize ioPTH monitoring to guide the extent of resection.

A reduction of more than 50% from the highest value recorded before surgical excision, within 10–15 min after removal of the hyperfunctioning tissue, is considered indicative of procedural success [12,60].

The primary indications for intraoperative PTH monitoring include:

• Confirmation of the removal of all hyperfunctioning parathyroid tissue without visualizing all glands
• Identification of inadequate PTH decline indicating the presence of additional hyperfunctioning glands
• Determination of the need for further exploration
• Differentiation between parathyroid and non-parathyroid tissues
• Bilateral internal jugular vein sampling to lateralize hyperfunctioning glands [60]

Blood samples for PTH measurement can be collected either peripherally by an anesthesiologist or directly from the internal jugular vein by the surgical team [61]. Baseline samples are typically drawn immediately before anesthesia induction or at skin incision. Additional samples are obtained at 5, 10, 15, and 20 min post-excision. Approximately 20–30% of patients may exhibit transient PTH spikes due to intraoperative manipulation. If PTH is not measured at the time of excision, these spikes may be misinterpreted, potentially leading to unnecessary bilateral exploration due to a false-negative decline in PTH [62].

Various criteria have been proposed to evaluate the success of surgery based on ioPTH dynamics, including the Miami, Halle, Rome, Vienna, and Charleston criteria [60]. The Miami criterion, the most widely used, was initially defined in 1993 as a >50% decline in PTH from baseline at 10 min post-excision [12]. It was later revised to indicate a >50% decrease from the highest pre-excision value [63]. This criterion has shown 97–98% accuracy in predicting postoperative normal blood calcium levels [64].

A recent study showed that ioPTH monitoring improved cure rates in 11% of patients with concordant ultrasound and scintigraphy, 11% with a single positive imaging modality, 33% with discordant imaging results, and 24% with negative or conflicting imaging results. Additional benefits were seen in 11% of patients undergoing minimally invasive parathyroidectomy (MIP), 24.6% with bilateral neck exploration (BNE), 12.4% during initial surgery, and 32.6% in reoperations [64]. In a large series, cure rates were 99.4% with ioPTH-guided focused surgery compared to 97.1% with BNE [63]. Intraoperative PTH monitoring helps confirm surgical cure in focused procedures and may predict multigland disease without visualizing all glands. MIP under ioPTH guidance is widely practiced, enhancing success rates and reducing the need for BNE [65]. However, in cases with concordant imaging, the use of ioPTH remains center-dependent. It may still be beneficial in patients with a single positive or discordant imaging result and is thus recommended where facilities are available [66]. With ioPTH monitoring, MIP can be performed safely with high success rates and lower morbidity compared to BNE.

Adjunct imaging modalities, such as gamma probe-guided surgery using 99mTc-sestamibi, add another valuable tool to the surgical armamentarium. This technique utilizes retained radiotracer uptake in hyperfunctioning parathyroid tissue. Protocols vary in terms of dosage and timing. In some centres, 20–25 mCi (740–925 MBq) of 99mTc-sestamibi is administered intravenously, followed by dual-phase scintigraphy within 3 h and same-day surgery [67]. Alternatively, sestamibi is administered again on the day of surgery [68], or 10–20 mCi is given 1–2 h prior to surgery [69]. Some protocols involve administering a low dose (1 mCi/37 MBq) just before surgery, guided by prior positive scintigraphy [70]. Radioactivity in parathyroid adenomas typically exceeds background levels by over 20%, a threshold used to confirm localization and reduce the need for further exploration, frozen section, or ioPTH monitoring [71]. However, gamma probes cannot reliably distinguish adenomas from hyperplasia and may not exclude multigland disease [72].

Ideal candidates for gamma probe-guided parathyroidectomy include patients undergoing first-time or redo surgery, those without nodular thyroid disease, and patients with confirmed single-gland disease on imaging [73]. Advantages include facilitation of focused surgery, reduced operative time, localization of ectopic glands, and intraoperative confirmation of success [74]. One center reported gamma probe suitability rates of 83%, with 10% for single adenomas and 50% for hyperplasia requiring BNE. Its use is debated in multigland disease, nodular goiter, or negative scintigraphy [75]. Contraindications include pregnancy, allergy to 99mTc-sestamibi, logistical constraints, and lack of benefit when abnormal glands are readily identifiable without the probe [76].

Colorimetric localization using intravenous methylene blue was first introduced by Dudley in 1971 [77]. While early studies demonstrated high staining rates (83–100%) in single gland disease, false-positive uptake in lymph nodes, thyroid, thymus, and adipose tissue was reported. Among patients with normal glands, staining occurred in 22–100%, and thyroid uptake was seen in 14.4% of cases [78]. Common side effects include blue discoloration of skin and urine, which are benign. Rarely, methylene blue can cause neurotoxicity, particularly in patients on serotonin reuptake inhibitors [78]. Routine use is not currently recommended outside of clinical trials [76].

Frozen section examination is commonly used to confirm that excised tissue is parathyroid. Although parathyroid tissue can typically be differentiated from other tissues, distinguishing it from thyroid tissue can be challenging. In a study of over 1500 frozen sections, accuracy was 99.2%, though misdiagnoses have been reported [79]. Frozen section cannot reliably differentiate adenoma from hyperplasia [76], and routine biopsies of all glands may increase the risk of hypoparathyroidism [80]. In secondary or tertiary hyperparathyroidism, frozen section may help verify remnant tissue or confirm suitability for autotransplantation or cryopreservation [81].

Emerging imaging technologies have been developed to enhance intraoperative localization and viability assessment of parathyroid glands, especially in thyroid surgery. These include autofluorescence imaging and indocyanine green (ICG) fluorescence, as parathyroid glands emit in the infrared spectrum [82]. Their fluorescence intensity exceeds that of surrounding tissues and remains visible post-resection, assisting with gland identification and confirmation [83,84], as visible in Figure 4.

Other novel methods under investigation include 5-aminolevulinic acid (5-ALA) fluorescence [85], optical coherence tomography (OCT) [86], laser speckle contrast imaging (LSCI) [83], dynamic optical contrast imaging (DOCI) [86], and Raman spectroscopy [87]. These techniques require further in vivo research to validate their clinical applicability and efficacy.

## 4. Surgical Outcomes

The outcomes of mediastinal parathyroidectomy are largely influenced by the adequacy of preoperative localization, the chosen surgical approach, and intraoperative monitoring. Minimally invasive techniques, such as video-assisted thoracoscopic surgery (VATS), have demonstrated significantly lower complication rates and shorter hospital stays compared to traditional open procedures, as reported in Figure 5. Studies report an average hospital stay of 3–4 days for VATS versus 7–10 days for sternotomy, with reduced postoperative pain and fewer respiratory complications [44,88].

Hypocalcemia is the most common postoperative complication, typically resulting from the abrupt decline in PTH levels following gland excision [89]. It may present as transient hypocalcemia, requiring temporary calcium and vitamin D supplementation, or—more rarely—as permanent hypoparathyroidism necessitating long-term management [89,90]. Routine postoperative monitoring of serum calcium and PTH levels is essential for early detection and management of hypocalcemia.

Persistent or recurrent hyperparathyroidism may arise if supernumerary glands are not identified preoperatively or if hyperfunctioning tissue is incompletely resected. Reported recurrence rates range from 5% to 10%, depending on the underlying pathology and the completeness of the initial surgical intervention.

### Redo Surgery

Redo surgery for mediastinal parathyroid disease is often necessary in cases of persistent or recurrent hyperparathyroidism. Unfortunately, literature indicates that redo surgery for mediastinal parathyroid glands (PTGs) is more common than exceptional. Reported reoperation rates range from 25% to 78% in three previous series [30,31,54].

In patients with multiple endocrine neoplasia type 1 (MEN1)—where subtotal or total parathyroidectomy with autotransplantation is typically recommended [91]—the incidence of persistent or recurrent hyperparathyroidism is particularly high. This may be attributed to the presence of supernumerary or ectopic glands, regrowth of remnant tissue, or hyperfunctioning autografts [92,93].

Interestingly, redo surgery for mediastinal PTGs may not be as technically demanding as reoperations in the cervical region. However, the complexity of these cases is often compounded by previous surgical interventions that distort normal anatomy and increase the risk of complications. Studies have shown that redo surgery mediastinal parathyroidectomy achieves higher success rates when guided by advanced preoperative imaging techniques, especially 4D-CT and PET/CT [24,25]. In cases with inconclusive or discordant imaging, selective venous sampling can be valuable for localizing hyperfunctioning tissue [32,33].

Careful surgical planning and the application of minimally invasive techniques have significantly improved outcomes in redo surgery cases, reducing the reliance on extensive open procedures. Nonetheless, such surgeries should be performed in specialized centers with multidisciplinary expertise in both endocrine and thoracic surgery to optimize outcomes and minimize morbidity [5].

## 5. Conclusions

Mediastinal parathyroid disease, though relatively rare, presents complex diagnostic and therapeutic challenges due to the variable location of ectopic glands and their proximity to critical thoracic structures. These factors make a standardized management approach impractical. Accurate preoperative localization is essential but must be adapted to each individual case, employing advanced imaging modalities, such as 4D-CT, PET/CT, and sestamibi SPECT/CT, according to the specific anatomical and clinical scenario to guide surgical planning and reduce morbidity.

Minimally invasive surgical techniques, particularly video-assisted thoracoscopic surgery (VATS) and robot-assisted thoracoscopic surgery (RATS), have transformed the landscape of mediastinal parathyroidectomy. However, the choice of approach must be customized based on gland location, prior surgeries, and patient-specific risks. Intraoperative adjuncts like ioPTH monitoring and gamma probe guidance are valuable tools, yet their use and interpretation also require tailoring to the case at hand to maximize precision and efficacy. Despite significant advances, recurrence remains a notable concern, especially in MEN1 patients or in cases involving supernumerary or deeply ectopic glands. Redo surgery, although increasingly effective with modern techniques, demands highly individualized planning and should be entrusted to experienced multidisciplinary teams in specialized centers.

In summary, optimal outcomes in mediastinal parathyroid disease are achieved through a tailored, case-specific strategy that integrates high-resolution imaging, intraoperative technologies, and the most appropriate minimally invasive surgical approach for each patient.

## Data Availability

Not applicable.

## Abbreviations

The following abbreviations are used in this manuscript:

APW	Aortopulmonary Window
BNE	Bilateral Neck Exploration
CT	Computed Tomography
DOCI	Dynamic Optical Contrast Imaging
EANM	European Association of Nuclear Medicine
ESRD	End-Stage Renal Disease
FNA	Fine-Needle Aspiration
ICG	Indocyanine Green
ioPTH	Intraoperative Parathyroid Hormone
IVC	Inferior Vena Cava
LSCI	Laser Speckle Contrast Imaging
MEN1	Multiple Endocrine Neoplasia Type 1
MIS	Minimally Invasive Surgery
MRI	Magnetic Resonance Imaging
MIP	Minimally Invasive Parathyroidectomy
OCT	Optical Coherence Tomography
PET/CT	Positron Emission Tomography/Computed Tomography
PHPT	Primary Hyperparathyroidism
PTH	Parathyroid Hormone
PTG	Parathyroid Gland
R/L	Right/Left
RATS	Robot-Assisted Thoracoscopic Surgery
SCa	Serum Calcium
SHPT	Secondary Hyperparathyroidism
SPECT	Single Photon Emission Computed Tomography
SVS	Selective Venous Sampling
99mTc	Technetium-99m
THPT	Tertiary Hyperparathyroidism
US	Ultrasound/Ultrasonography
VATS	Video-Assisted Thoracoscopic Surgery
